# Futility Monitoring in Clinical Trials

**DOI:** 10.1002/sim.70157

**Published:** 2025-06-11

**Authors:** Ana M. Ortega‐Villa, Megan C. Grieco, Kevin Rubenstein, Jing Wang, Michael A. Proschan

**Affiliations:** ^1^ Office of Biostatistics Research National Institute of Allergy and Infectious Diseases Bethesda Maryland USA; ^2^ Axle Informatics Bethesda Maryland USA; ^3^ Clinical Monitoring Research Program Directorate Frederick National Laboratory for Cancer Research Frederick Maryland USA

**Keywords:** beta spending functions, conditional power, futility monitoring, predicted interval plots, predictive power, stochastic curtailment

## Abstract

At the beginning of a phase III clinical trial, there is great optimism. After all, the phase II trial results were encouraging. Then, early data from the phase III trial trend in the wrong way, but there is still an opportunity for the trend to reverse and become statistically significant by the end. At what point does optimism become denial of reality? How do we decide when a clinical trial is futile? What does futility even mean? This tutorial reviews different concepts and tools for evaluating futility, including conditional and predictive power, reverse conditional power, predicted interval plots, revised unconditional power, and beta spending functions.

## 1 Introduction

Randomized controlled trials are the highest level of medical evidence, but they can go wrong in many ways: Recruitment could be poor, participants might feel over‐burdened by trial procedures and drop out, the event rate may have been seriously overestimated, or treatment adherence may be poor. Consequently, the original trial question cannot be answered with reasonable power. Factors like low recruitment, high dropout, lack of adherence, and so forth, lead to *operational futility*, the inability to determine whether an intervention works. For example, investigators in the ACTIV‐4B trial [1] planned to evaluate the effect of anticoagulant and antiplatelet therapy among symptomatic, clinically stable outpatients with COVID‐19. They anticipated a control event rate of between 4% and 8% for their composite primary endpoint of mortality, symptomatic venous/arterial thromboembolism, myocardial infarction, stroke, or hospitalization for cardiovascular or pulmonary cause. After 558 patients initiated treatment, only five patients among the four arms experienced the primary endpoint. It was clear that the final number of events would yield insufficient power to make reliable conclusions, and the trial was stopped.

Alternatively, treatment may be much less effective than originally thought. This is a very different kind of futility because the question can be answered reliably, and the answer is that the intervention does not work. This was the case with the HIV vaccine trial of AIDSVAX in 5108 men who have sex with men and 309 women at high risk for acquiring HIV [2]. There was only a 6% vaccine efficacy (p=0.59). In some cases, such as when a treatment is already being used even though no randomized trial has demonstrated efficacy on the important clinical endpoint, one may want to continue the trial to demonstrate that the treatment does not work. For example, only strong evidence from the Cardiac Arrhythmia Suppression Trial [3] could convince doctors that suppressing cardiac arrhythmias in patients with a prior heart attack using a certain class of drugs was harmful.

Two questions should be considered when trying to decide whether to stop a trial for futility: (1) Is a null result likely? (2) Would a null result still be meaningful? By “null result”, we mean a non‐statistically significant result at the appropriate type I error rate. If the answer to the first question is no, then the trial is likely to have a statistically significant result, and there would be no reason to stop for futility. If the answer to the first question is yes, then the second question may come into play. It tells us whether the null result we are likely to see reliably tells us that treatment does not work. Most futility methods address the first question. For example, *conditional power* is the conditional probability that the result at the end of the trial will be statistically significant, given the current results and a projection of future data. Low conditional power means that a null result is likely. We argue that the calculation of *revised unconditional power*, namely re‐doing the original power calculation but using revised estimates of nuisance parameters, is a useful tool for answering the second question. Whichever futility tools are used should be pre‐specified in the protocol. Pre‐planning is essential to maintain the integrity and reproducibility of research.

## 2 Review of Information, Z‐Scores, and B‐Values

### 2.1 Special Setting

We give a brief review and heuristic view of information, z‐scores, and B‐values. More details can be found in Lan and Zucker [4], Chapter 2 of Proschan et al. [5], and Chapters 3 and 11 of Jennison and Turnbull [6].

We begin with the continuous outcome setting in which each patient receives both treatments in random order. The outcome variable $D_{i}$ for patient i is the paired difference between treatment and control, and assume for simplicity that $D_{1},D_{2},\ldots$ are iid. N(δ,1), where $\delta =E(D_{i})$ is the treatment effect parameter. Let (n,N), $\left({\hat{\delta }}_{n},{\hat{\delta }}_{N})=(S_{n}/n,S_{N}/N\right)$, and $\left(Z_{n},Z_{N})=(S_{n}/\sqrt{n},S_{N}/\sqrt{N}\right)$ be the respective sample sizes, sample means, and z‐scores at the interim and final analysis. In this simple setting, the sample size is a good measure of the amount of information in our estimator. The sample size ratio, t=n/N, is called the *information fraction* (or *information time*) because it measures the fraction of information contained in ${\hat{\delta }}_{n}$. Note that t=0 at the beginning (n=0), and t=1 at the end of the trial (n=N). We denote the z‐score at information fraction t by Z(t). To control the type I error rate when we monitor at information fractions $t_{1},\ldots ,t_{k}$, we must determine the joint distribution of $Z(t_{1}),\ldots ,Z(t_{k})$.

#### 2.1.1 B‐Values

There are advantages to monitoring using a transformation of the z‐score called the B‐value. To motivate this transformation, consider the calculation of conditional power, the conditional probability of a significant result at the end of the trial, given the current data (summarized by the sufficient statistic $S_{n}$). For a one‐sided test at the 0.025 significance level, the conditional power is:

(1) $$\begin{array}{ll} CP & =P\left(\frac{S_{N}}{\sqrt{N}}>1.96\;|\;S_{n}=s\right)\\ & =P\left(\frac{S_{n}+S_{N}-S_{n}}{\sqrt{N}}>1.96\;|\;S_{n}=s\right)\\ & =P\left(\frac{s+S_{N}-S_{n}}{\sqrt{N}}>1.96\;|\;S_{n}=s\right)\\ & =P\left(\frac{s+S_{N}-S_{n}}{\sqrt{N}}>1.96\right)\\ & =P\left(\frac{s}{\sqrt{N}}+\frac{S_{N}}{\sqrt{N}}-\frac{S_{n}}{\sqrt{N}}>1.96\right) \end{array}$$

The fourth line follows from the fact that $S_{N}-S_{n}$ and $S_{n}$ are sums of non‐overlapping components, and are therefore independent (sums have *independent increments*). Because the denominator of the last line of Expression (1) is $N^{1/2}$, we define the *B‐value* B(t) as

$$B(t)=\frac{S_{n}}{\sqrt{N}},\;\text{for}\;t=n/N,\;n=1,\ldots ,N$$

We define $S_{0}=0$ and B(0)=0. Also, $B(1)=B(N/N)=S_{N}/N^{1/2}$ is the z‐score at the end of the trial. The observed B‐value at the interim analysis with $S_{n}=s$ is $b=s/N^{1/2}$. We can express Equation (1) in terms of B‐values as

(2) $$\begin{array}{ll} P\left(\frac{s}{\sqrt{N}}+\frac{S_{N}}{\sqrt{N}}-\frac{S_{n}}{\sqrt{N}}>1.96\right) & =P\left\{b+B(1)-B(t)>1.96\right\}\\ & =P\left\{B(1)-B(t)>1.96-b\right\} \end{array}$$

B‐values inherit the independent increments property from the corresponding sums. That is, for $t_{1}<t_{2}<\cdots <t_{k}$, $B(t_{1}),B(t_{2})-B(t_{1}),\ldots ,B(t_{k})-B(t_{k-1})$ are independent. Other properties of B‐values can be derived (see appendix), namely

B1: B(t)∼N(mean=θt,var=t), where θ=E{B(1)}=E{Z(1)} is the expected value of the z‐score at the end of the trial, known as the *drift parameter*.
B2: For $t_{1}\leq t_{2}$, $B(t_{2})-B(t_{1})∼N(\text{mean}=\theta (t_{2}-t_{1}),\mathrm{var}=t_{2}-t_{1})$.

From property B2 with $t_{1}=t$ and $t_{2}=1$, (2) simplifies to the general formula for conditional power:

(3) $$CP=1-\Phi \left(\frac{1.96-b-\theta (1-t)}{\sqrt{1-t}}\right)=\Phi \left(\frac{b+\theta (1-t)-1.96}{\sqrt{1-t}}\right)$$

where Φ denotes the standard normal distribution function.

The connection between B‐values and z‐scores is

$$B(t)=\frac{S_{n}}{\sqrt{N}}=\sqrt{\frac{n}{N}}\frac{S_{n}}{\sqrt{n}}=\sqrt{t}\;Z(t)$$

from which we can deduce the following properties of z‐scores:

Z1: $Z(t)∼N(\text{mean}=\theta \sqrt{t},\mathrm{var}=1),\;\text{where}\;\theta =E\{Z(1)\}$.
Z2: For $t_{1}\leq t_{2}$, $\text{cov}\{Z(t_{1}),Z(t_{2})\}=\sqrt{t_{1}/t_{2}}$.

### 2.2 General Setting

We now consider the general setting of estimating some treatment effect parameter δ. Examples of δ include a difference in means or proportions, or a log hazard ratio, depending on whether the primary endpoint is continuous, binary, or survival. Let $\hat{\delta }$ be an estimator of δ with standard error $\sigma_{\hat{\delta }}$, and assume that sample sizes are large enough to treat $\sigma_{\hat{\delta }}$ as a known constant. The z‐statistic is

(4) $$Z=\frac{\hat{\delta }}{\sigma_{\hat{\delta }}}$$

In the single mean setting of Section 2.1, our estimator, ${\hat{\delta }}_{n}$, was a sample mean of n N(δ,1) observations and the sample size, n, was taken as a measure of information in ${\hat{\delta }}_{n}$. The variance of ${\hat{\delta }}_{n}$ is 1/n, the reciprocal of the information. In other words, in the single mean setting, the amount of information was the reciprocal of the variance of the estimator. We use this same definition in the general setting. The *information*, I, in estimator $\hat{\delta }$ is defined by

$$I=1/\mathrm{var}(\hat{\delta })$$

Even though $\hat{\delta }$ might be generated from a survival analysis, for example, $\hat{\delta }$ behaves like a sample mean of I N(δ,1) observations in the sense that $\mathrm{var}(\hat{\delta })=1/I$, just like it would be if $\hat{\delta }$ were a sample mean of I N(δ,1) observations. Likewise, the “sum” defined by $S_{I}=I\hat{\delta }$ behaves like the sum of I iid. N(δ,1) observations in the sense that $\mathrm{var}(S_{I})=I$ and $S_{I}$ has independent increments. We define the *information fraction* (or *information time*) at an interim analysis with information I as

(5) $$t=I/I_{\text{end}}$$

the ratio of the information at the interim analysis to the information at the end of the trial. In the iid. N(δ,1) setting of Section 2.1, Formula (5) reduces to n/N. As in Section 2.1, we define the B‐value B(t) at information time t as the “sum”, $S_{I}=I\hat{\delta }$, divided by the standard deviation, $I_{\text{end}}^{1/2}$, of the sum at the end of the trial:

$$B(t)=\frac{S_{I}}{\sqrt{\mathrm{var}(S_{I_{\text{end}}})}}=\frac{S_{I}}{\sqrt{I_{\text{end}}}}=\sqrt{\frac{I}{I_{\text{end}}}}\frac{S_{I}}{\sqrt{I}}=\sqrt{t}Z(t)$$

Joint distributions of z‐scores and B‐values are the same as in the $D_{i}$ iid. N(δ,1) case of Section 2.1, except that information time is now defined as $t=I/I_{\text{end}}$, the ratio of current information, $I=1/\mathrm{var}(\hat{\delta })$, to the final information, $I_{\text{end}}=1/\mathrm{var}({\hat{\delta }}_{\text{end}})$.

 

Properties B1, B2, Z1, and Z2 from Section 2.1 continue to hold in more general settings, with information fraction t defined as (5) and θ defined as the expected z‐score at the end of the trial. The information fraction is approximated by the ratio of interim to final sample sizes in non‐survival settings and the ratio of interim to final number of patients with events in survival settings.

Just remember that, under regularity conditions:

1. $\hat{\delta }$ and $S_{I}=I\hat{\delta }$ behave like a sample mean and sum of I iid. N(δ,1) random variables.
2. The B‐value B(t) behaves like $S_{I}/\sqrt{I_{\text{end}}}$.

## 3 Question 1: Is a Null Result Likely?

### 3.1 Conditional Power

Conditional power (CP) is a useful statistical tool that can inform the decision on whether to stop a trial for futility and is defined as the conditional probability of reaching a statistically significant treatment benefit by the end of the trial, given current results. CP must be computed by a person who has access to the results by arm (i.e., an unblinded statistician). Low CP means that a null result at the end of the trial is likely, suggesting that we may want to stop for futility. Conditional power is usually computed under different assumptions about the treatment effect. Three common choices are: (1) the originally hypothesized effect, (2) the currently observed effect, or (3) something between the originally hypothesized and currently observed effects.

**FIGURE 1:**
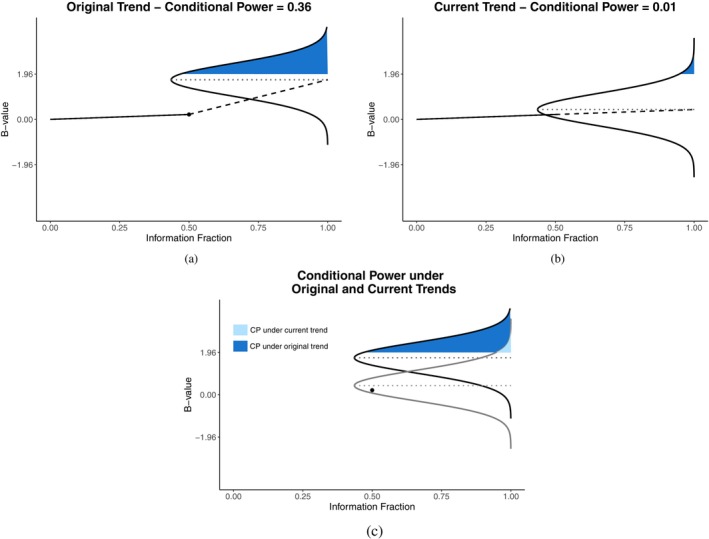
Graphical representation of conditional power for a trial with 85% power with no monitoring, an interim analysis at information fraction 50% with an interim z‐score of 0.3. Panels (a) and (b) are for CP computed under the originally hypothesized treatment effect and current trend, respectively, and panel (c) shows both in one graph.

We derived Formula (3) for CP for the specific setting of Section 2.1, but that formula is equally valid whenever the Brownian motion paradigm applies. Figure 1 is a graphical representation of CP for a trial with 85% power (with no monitoring), an interim analysis at information fraction 50%, and an interim z‐score of Z(0.5)=0.3 (B‐value $0.3\sqrt{0.5}=0.212$). Panels (a) and (b) correspond to computing CP under the originally hypothesized effect and the current effect, respectively, while panel (c) shows both in a single visual. Graphically, Equation (3) for CP corresponds to the following steps: (1) draw a line segment with slope θ (the drift parameter) from the current information time and B‐value, (t,B(t))=(0.5,0.212), to the end of the trial at t=1; (2) use the line segment value at t=1 as the mean and use 1−t as the variance of a normal distribution; (3) compute conditional power as the area to the right of $Z_{\alpha /2}$ under the normal distribution obtained in step 2.

Under the originally hypothesized treatment effect, the drift parameter θ is 1.96+0.84=2.80, 1.96+1.04=3.00, or 1.96+1.28=3.24 for 80%, 85%, or 90% power, respectively. Because our example has 85% power, θ=3 under the originally hypothesized treatment effect. Recall that B(t) has mean θt, so the current effect estimate of θ is B(t)/t=B(0.5)/0.5=0.212/0.5=0.424. The y‐value at t=1 for the dashed line segment in Figure 1 is B(0.5)+θ(1−t), which is what we now expect the z‐score to be at the end of the trial, given the data so far. CP is the shaded area above 1.96 under the superimposed normal curve. In our example depicted in Figure 1, panels (a) and (b) show that CP=0.36 and CP=0.01 under the originally hypothesized treatment effect and the current trend, respectively. The main CP calculation should be performed under the originally assumed treatment effect. The CP calculated under the current trend is highly variable and, in some cases—particularly early in a trial—may be misleading.

*Stochastic curtailment* [7] is a rule that stops a trial for futility if CP drops below a threshold Γ. Usually Γ≤0.2. Lan et al. [7] show that if a trial is monitored continuously, power using the stochastic curtailment rule that stops if CP calculated under the originally hypothesized treatment effect is ≤Γ, the type II error rate is β/(1−Γ), where β is the type II error rate with no monitoring. For example, suppose a trial has 90% power with no monitoring (β=0.1) and stops for futility if CP under the originally hypothesized effect ever drops below Γ=0.2. Even with continuous monitoring, the type II error rate is only 0.10/(1−0.20)=0.125 (power 0.875). The type II error rate is very close to 0.10 for a more typical monitoring schedule.

The stochastic curtailment bound of Lan et al. [7] does not apply when computing CP under the current trend. Table 1 presents the type II error rate from declaring futility whenever conditional power under the current trend drops below 0.20 for a trial with varying numbers of looks and unconditional power of 80%, 85%, and 90%. For example, suppose a trial has 90% power with no monitoring, and uses a stochastic curtailment rule that stops if CP under the current trend is ≤0.20 at any of 6 looks equally spaced in terms of information. Then the actual type II error rate is 0.306 (power 0.694). This is clearly an unacceptable loss of power! For this reason, we emphasize that the primary conditional power calculation should be under the originally hypothesized treatment effect.

**TABLE 1:** Type II error rate from declaring futility whenever conditional power under the current trend drops below 0.20.

	Number of looks					
Unconditional power	1	2	3	4	5	6
0.80	0.200	0.259	0.312	0.356	0.392	0.422
0.85	0.150	0.204	0.256	0.300	0.337	0.368
0.90	0.100	0.147	0.196	0.238	0.275	0.306

We have developed the Conditional Power Shiny Web Application to calculate conditional power for binary, continuous, and time‐to‐event endpoints.

### 3.2 Example: LUME‐Lung 2 Trial

The LUME‐Lung 2 Trial randomized patients with pre‐treated non‐squamous non‐small cell lung cancer to nintedanib or placebo on top of pemetrexed [8]. The primary endpoint was centrally adjudicated progression‐free survival (PFS), analyzed using a stratified logrank test, and the anticipated 713 events would provide 90% power to detect a hazard ratio of 0.78. There were two planned analyses, at information fractions t=0.5 and t=1. The Lan‐DeMets [9] spending function corresponding to boundaries similar to those of O'Brien and Fleming [10] was used for efficacy, and the non‐binding futility guideline considered stopping for futility if conditional power under the current trend estimate dropped below 0.20. Importantly, the interim futility analysis used investigator‐assessed, not centrally adjudicated, PFS because the latter was available only for those experiencing progressive disease.

On 3/14/2011, with 346 events (t=0.485), the data and safety monitoring board recommended stopping enrollment because of futility. Conditional power under the current trend was 0.103, below the futility threshold of 0.20. Enrollment stopped on 6/18/2011, and the placebo was discontinued on 7/29/2011. Subsequently, on 7/9/2012, the database was locked for the primary analysis; 353 nintedanib and 360 placebo patients had been randomized, and there were 498 PFS events by central review. The hazard ratio was 0.83 with a 95% confidence interval (0.70,0.99), and the p value was p=0.0435. In other words, although the trial had been declared futile on 3/14/2011, results on 7/9/2012 showed a statistically significant benefit!

Lesaffre et al. [11] conducted retrospective analyses to identify how such a reversal could happen. They examined results for both investigator‐assessed and central review after approximately 10%, 20%, 30%, 40%, 50%, 60%, and 70% of events had accrued. They noted that conditional power results were quite variable, dropping below 0.20 only at the actual interim analysis time for investigator‐assessed PFS. Lesaffre et al. [11] identified several possible explanations for the conflicting results of LUME‐Lung 2, including differences between centrally‐ and investigator‐assessed PFS, confounding factors and imbalances, and chance. They recommended basing futility decisions on more than one endpoint and suggested that a lower conditional power threshold may have been preferable. They also noted that “…early monitoring for lack of benefit can be unreliable as insufficient events may have been observed to produce a reliable effect.” We believe that this latter point is very important. It can be shown that the actual type II error from applying a conditional power threshold of 0.20 using the current trend estimate after 10%, 20%, 30%, 40%, 50%, 60%, 70%, and 100% information is approximately 0.40 instead of the intended level of 0.10. Such an inflated rate is unacceptable. On the other hand, the type II error rate from using a conditional power threshold of 0.20 under the original hypothesis after 10%, 20%, 30%, 40%, 50%, 60%, and 70% of events is approximately 0.103.

With respect to the actual interim analysis at t=0.485, the threshold of 0.20 would not have been close to being crossed had conditional power been computed under the original hypothesis. Specifically, from the interim hazard ratio of 0.92 and conditional power value 0.103 from Hanna et al. [8] and Lesaffre et al. [11], we deduce that the interim z‐score, parameterized such that a positive z‐score implies benefit, is approximately 0.733. Therefore, conditional power under the original hypothesis corresponding to 90% power is approximately

$$\Phi \left(\frac{0.733\sqrt{0.485}+3.24(1-0.485)-1.96}{\sqrt{1-0.485}}\right)=0.620$$

nowhere near the threshold of 0.20. The LUME‐Lung 2 trial illustrates the problem with applying stochastic curtailment to the current estimate of the treatment effect.

### 3.3 Reverse Conditional Power

**FIGURE 2:**
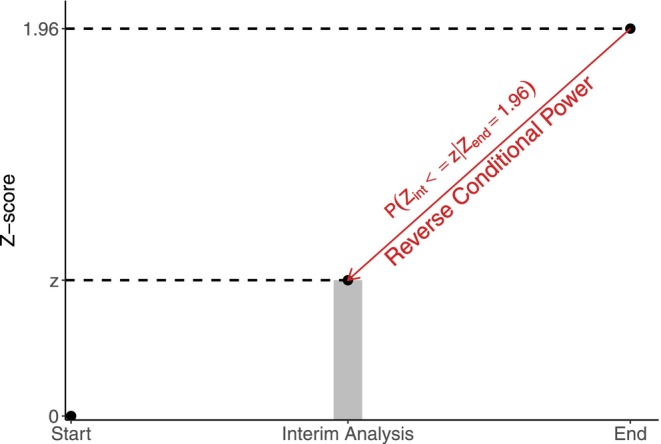
Graphical representation of reverse conditional power. The arrow indicates we are starting at the end of the trial and projecting to the monitoring time.

*Reverse conditional power (RCP)* [12] is an alternative tool used to inform on the futility of a clinical trial. In CP, we calculate the conditional probability of obtaining a statistically significant result at the end of the trial, given the current result (i.e., $P(Z_{\text{end}}\geq 1.96\;|\;Z_{\text{int}}=z)$ when the one‐sided α is 0.025). In RCP, we calculate the conditional probability of obtaining results at least as disappointing as the current result, given that we will obtain a (barely) statistically significant result at the end of the trial (i.e., $P(Z_{\text{int}}\leq z\;|\;Z_{\text{end}}=1.96)$ when the one‐sided α is 0.025). Another way to think about RCP is as the proportion of trials that would see results as bad or worse than the current result among those trials that eventually (barely) reach a statistically significant benefit. Figure 2 presents a graphical representation of RCP.

Similarly to how we use CP, we stop a trial for futility if RCP is low. The concept of stochastic curtailment can also be applied to RCP, and is called *reverse stochastic curtailment* [12]. One attractive option stops for futility if RCP≤0.025.

The main advantage of using RCP instead of CP is that no assumptions need to be made about the treatment effect because RCP conditions on $Z_{\text{end}}$, which is a sufficient statistic [12]. Thus, RCP avoids the problem seen in the LUME‐Lung 2 Lung Trial from calculating CP under the current trend estimate of the treatment effect. Not having to estimate the treatment effect results in RCP having a low probability of a discrepant result for monitoring versus no monitoring. Consequently, the loss in power from using RCP is very low.

Table 2 confirms the small loss of power from reverse stochastic curtailment using a threshold of 0.025. The table shows the type II error rates from stopping for futility whenever RCP drops below 0.025 for a trial with different numbers of equally spaced looks and different powers. Note that there is minimal inflation of the type II error rate under all considered combinations of power and number of looks.

**TABLE 2:** Type II error rate from stopping for futility whenever reverse conditional power drops below 0.025.

	Number of looks					
Unconditional power	1	2	3	4	5	6
0.80	0.200	0.203	0.206	0.209	0.212	0.214
0.85	0.150	0.153	0.156	0.159	0.161	0.163
0.90	0.100	0.102	0.105	0.107	0.110	0.112

The formula for RCP can be derived using the fact that, for bivariate normal random variables (X,Y) with means $\left(\mu_{X},\mu_{Y}\right)$, variances $\left(\sigma_{X}^{2},\sigma_{Y}^{2}\right)$, and correlation ρ, the conditional distribution of X given that Y=y is normal with mean $\mu_{X}+\rho (\sigma_{X}/\sigma_{Y})(y-\mu_{Y})$ and variance $\sigma_{X}^{2}(1-\rho^{2})$. Apply this formula to X=B(t) and Y=B(1), which are bivariate normal with respective means (θt,θ), respective variances (t,1), and correlation $\rho =\sqrt{t}$:

(6) $$\begin{array}{ll} & \left\{B(t)\;|\;B(1)=1.96\right\}\\ & \;∼N\left\{\text{mean}=\theta t+\sqrt{t}\left(\frac{\sqrt{t}}{\sqrt{1}}\right)(1.96-\theta );\;\mathrm{var}=t(1-t)\right\}\\ & \;∼N\left\{\text{mean}=1.96t;\mathrm{var}=t(1-t)\right\}\\ & \text{RCP}=P\left\{B(t)\leq z\sqrt{t}\;|\;B(1)=1.96\right\}=\Phi \left\{\frac{z\sqrt{t}-1.96t}{\sqrt{t(1-t)}}\right\} \end{array}$$

We illustrate the use of Formula (6) to compute reverse conditional power in an example with an interim look at t=1/2 and an observed z‐score of Z(1/2)=z=0. Substituting these values into Formula (6) results in RCP=Φ(−1.96)=0.025, which just meets the reverse stochastic curtailment threshold, RCP≤0.025. Contrast this with the stochastic curtailment rule that stops if CP under the original hypothesis is ≤0.20. If the trial had power 0.85, then θ=1.96+1.04=3 and, from Formula (3) with t=1/2 and $b=z\sqrt{1/2}=0$, conditional power is

$$\text{CP}=\Phi \left(\frac{0+3(1-1/2)-1.96}{\sqrt{1-1/2}}\right)=0.258$$

This would not meet the CP≤0.20 criterion. Interestingly, if the trial had been powered at 0.80 instead of 0.85, then θ=1.96+0.84=2.80 and CP=0.214. This is very close to meeting the CP≤0.20 threshold. Still, the threshold for stopping for futility, CP≤0.2, would not have been met, while the threshold for RCP would have been met.

In the LUME‐Lung 2 trial example in Section 3.1, reverse conditional power at the actual interim analysis at t=0.485 is

$$\Phi \left(\frac{0.733\sqrt{0.485}-1.96(0.485)}{\sqrt{0.485(1-0.485)}}\right)=0.189$$

which is not close to the threshold of 0.025, and therefore, the trial would not have been stopped for futility had RCP been used as the stopping rule.

We have developed the Reverse Conditional Power Shiny Web Application to calculate reverse conditional power.

### 3.4 Predicted Interval Plots

#### 3.4.1 Predicted Intervals

Evans et al. [13] proposed predicted intervals as a monitoring tool. Predictive intervals combine observed data from the trial and simulated future data to predict the confidence interval for the parameter of interest at the end of the trial.

Simulation of unobserved data at the time of the calculation can be performed under reasonable assumptions, as sensitivity analyses. For example, calculations might use the observed trend, the null hypothesis, and one (or more) alternative hypotheses. Predicted intervals can be calculated for binary, continuous, and time‐to‐event endpoints. Below, we present an example of the calculation of predicted intervals for a binary endpoint as presented by Evans et al. [13].

Let $N_{i}$ and $N_{c}$ denote the total targeted sample sizes for the intervention and control arms, respectively. Let $p_{i}$ and $p_{c}$ denote the proportions of events after combining observed and simulated data for the intervention and control arms, respectively. The predicted 100(1−α) percent interval is given by:

$$(p_{c}-p_{i})\pm z_{\alpha /2}\sqrt{\frac{p_{c}(1-p_{c})}{N_{c}}+\frac{p_{i}(1-p_{i})}{N_{i}}}$$

Predicted intervals allow the user to gather information about the effect size of interest, gauge sensitivity of effect estimates to various assumptions about unobserved data (e.g., generated under the null vs. alternative hypothesis), and assess efficiency gains (in terms of reduction of the width of the interval) by continuing enrollment.

For time‐to‐event endpoints, Evans et al. [13] suggest considering different types of censoring (e.g., due to loss‐to‐follow‐up vs. censoring due to timing of the interim look), as well as generating unobserved event times based on assumptions about the distribution of event times. Alternatively, to avoid making assumptions about this distribution, one can use an approximation using the observed Z‐score of the logrank test and the B‐value formulation. At the interim analysis one can calculate information time t=d/D, where d and D are the observed and targeted number of events, respectively, the interim z‐score $z_{\text{interim}}$ and associated B‐value $b=\sqrt{t}z_{\text{interim}}$. Instead of simulating the unobserved data, we simulate the B‐value/Z‐score at the end of the trial:

$$B(1)=Z(1)=b+\theta (1-t)+N(0,\text{sd}=\sqrt{1-t})$$

Our estimate of θ can be obtained using the observed trend or using a hypothesized value of the hazard ratio (HR).

• Observed trend: θ=b/t
• Hypothesized trend: Will depend on the randomization scheme.
  · 1:1 randomization $\to \theta \approx \ln(HR)\sqrt{D/4}$
  · 2:1 randomization $\to \theta \approx \ln(HR)\sqrt{2D/9}$

Once B(1) is computed, one can estimate the lower and upper limits of the interval as $B(1)\pm z_{\alpha /2}$. We can then recover the estimate of the hazard ratio and respective confidence intervals from the above.

#### 3.4.2 Predicted Interval Plots

Li et al. [14] propose predicted interval plots (PIPS), a visualization tool that summarizes results from repeated simulations of predicted intervals, and recommend constructing PIPS under different assumptions for the data‐generating mechanism of unobserved data. The procedure is as follows:

1. Make an assumption about the data‐generating mechanism. Examples include the observed trend, the null hypothesis, and the alternative hypothesis.
2. Obtain M (a large number; in the example, we use 500) predicted point estimates and associated predicted intervals for a given coverage probability (we used 95%).
3. Plot predicted point estimates using solid dots and their corresponding prediction intervals as solid lines through the dots.
4. Li et al. [14] propose grouping estimates and using the brightness of horizontal lines to denote values that are closest to a statistic of interest, such as the mean or median value over multiple simulations. In our example, we use the median.
  - Li et al. [14] propose that the grouping be done using values of the estimated conditional probability density function (CPDF) obtained by kernel density estimation, given the observed interim data. The mode can be extracted by obtaining the point estimate with the highest estimated CPDF value, and using brighter colors for estimates closest to the mode.
  - Alternatively, we could use percentiles of the point estimate distribution and use brighter colors for estimates closest to the median. This approach is illustrated in our example.
5. Indicate the null value of the parameter of interest using a vertical line on the plot.

**FIGURE 3:**
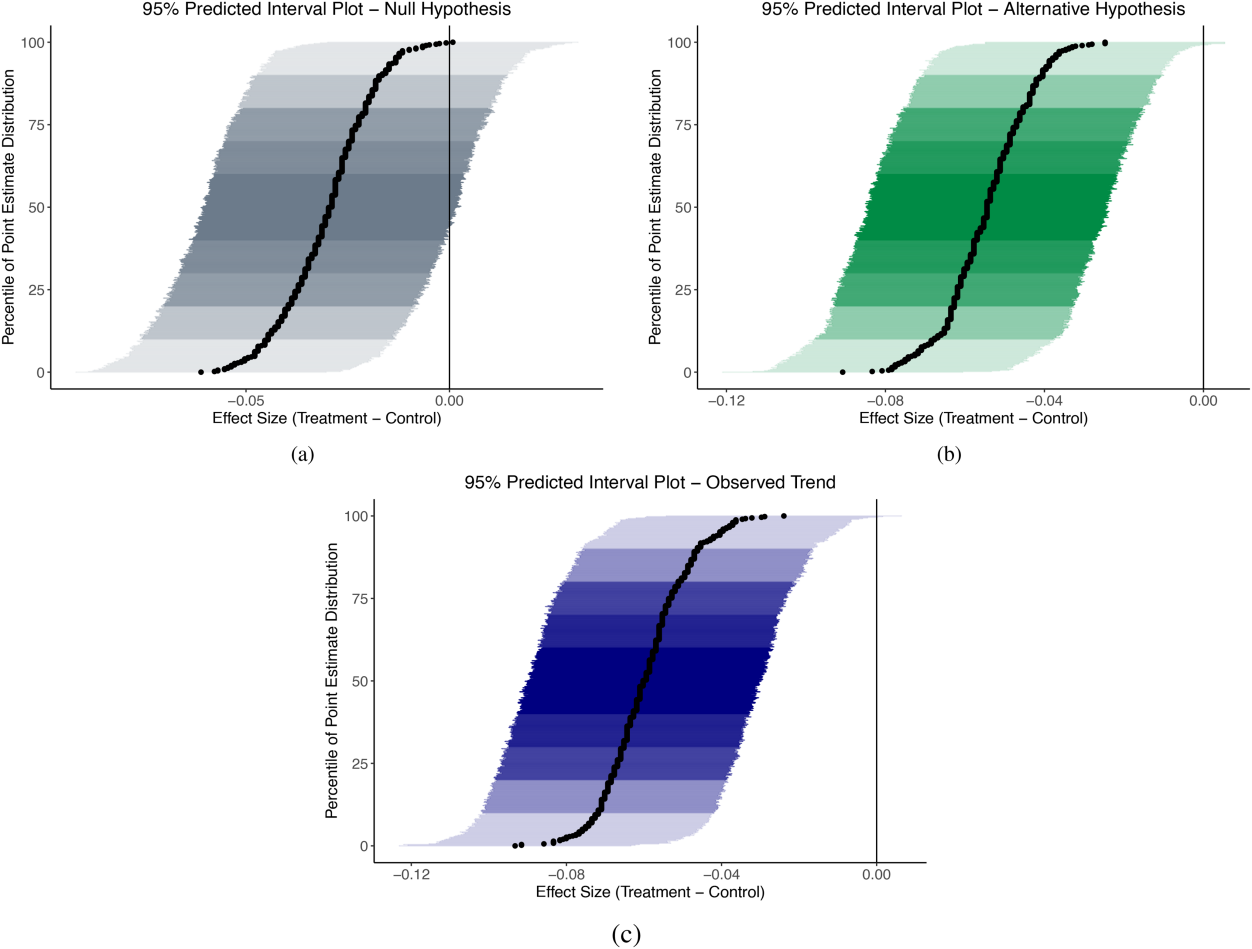
Example of predicted interval plots for a trial with $\pi_{i}=0.15$, $\pi_{c}=0.2$, total per‐arm sample sizes of $N_{c}=N_{i}=1212$, and an interim analysis when 606 participants in each arm have information. Subscripts i and c denote intervention and control arms, respectively. Panels (a), (b), and (c) present predicted intervals under the null and alternative hypotheses and the current trend, respectively.

Consider a trial in which the probabilities of events in the intervention and control arms are $\pi_{i}=0.15$ and $\pi_{c}=0.2$, the sample sizes at the end of the trial are $N_{c}=N_{i}=1212$, and we conduct an interim analysis when 606 participants in each arm have information. There are 123 and 90 events in the control and intervention arms, respectively. Figure 3 presents PIPS under the null ($\pi_{c}=\pi_{i}=0.2$—panel a) and alternative ($\pi_{i}=0.15$ and $\pi_{c}=0.2$—panel b) hypotheses, as well as under the current trend (panel c). Note that the PIPS under all trends support continuation of the trial. Even assuming the null hypothesis for future data, most final intervals are entirely to the left of the vertical line at the null hypothesis value of 0.

Further, Table 3 presents the observed 95% confidence interval of the point estimate at information time = 0.5, and predicted intervals (at the median of the distribution) under the null and alternative hypotheses, and the observed trend. We see that the width of the interval decreases between 27%–29.4% depending on the data‐generating mechanism.

**TABLE 3:** Example of predicted intervals (PI) for a trial with $\pi_{i}=0.15$, $\pi_{c}=0.2$, total per‐arm sample sizes of $N_{c}=N_{i}=1212$, and an interim analysis when 606 participants in each arm have information. Subscripts i and c denote intervention and control arms, respectively. The observed confidence interval in row 1 represents the 95% confidence interval at information time 0.5, and predicted intervals are under the null and alternative hypotheses, and under the observed trend.

Interval	Estimate	Lower	Upper	Interval width
Observed confidence interval	−0.059	−0.102	−0.017	0.085
PI under null	−0.026	−0.057	0.005	0.062
PI under alternative	−0.050	−0.081	−0.020	0.061
PI under observed trend	−0.056	−0.086	−0.026	0.060

We have developed the Predicted Interval Plots Shiny Web Application to calculate and graph prediction intervals for binary, continuous, and time‐to‐event endpoints.

### 3.5 Beta Spending Functions

Alpha spending functions are the most common tools for monitoring for benefit, but the same idea can be used to monitor for futility. We choose a function $\beta_{*}(t)$, 0≤t≤1, specifying the rate at which type II error rate (1−power) is spent, with $\beta_{*}(0)=0$ and $\beta_{*}(1)=\beta$, the total intended type II error rate. Lower boundaries $l_{1},\ldots ,l_{k}$ are chosen such that, under the pre‐specified alternative hypothesis, the cumulative probability of making a type II error by information time $t_{j}$ is $\beta_{*}(t_{j})$. Keep in mind that to make a type II error, we must cross the lower boundary without having first crossed the upper boundary $u_{1},u_{2},\ldots$ for benefit. Therefore, the cumulative type II error rate by information time $t_{j}$ is

(7) $$P_{\theta }(\text{cross}\;l_{1},\ldots ,l_{j}\;\text{without first crossing}\;u_{1},\ldots ,u_{j},)$$

where this probability is computed under the drift parameter θ. We want (7) to be $\beta_{*}(t_{j})$.

It is computationally easier to think in terms of the *first crossing probability (FCP)*, namely the probability of making a type II error for the first time at information time $t_{j}$. To make a type II error for the first time at $t_{j}$, we must not have crossed a lower or upper boundary previously. Thus,

(8) $$FCP(t_{j})=P_{\theta }\left\{l_{1}\leq Z(t_{1})\leq u_{1},\ldots ,l_{j-1}\leq Z(t_{j-1})\leq u_{j-1},Z(t_{j})<l_{j}\right\}$$

Equating (8) to $\beta_{*}(t_{j})-\beta_{*}(t_{j-1})$ ensures that the cumulative type II error rate by $t_{j}$ is

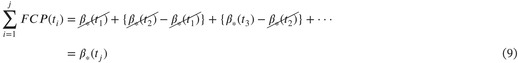

Before solving Equation (8) for $l_{1},\ldots ,l_{k}$, we impose one additional requirement that the final lower boundary, $l_{k}$, should equal the final upper boundary, $u_{k}$. That way, there is an unambiguous conclusion at the end: Benefit if $Z(1)\geq u_{k}$ and no benefit if $Z(1)<l_{k}=u_{k}$. We search over a grid of values of the drift parameter. For each such value, we solve for $l_{1},\ldots ,l_{k-1}$ by equating (8) to $\beta_{*}(t_{j})-\beta_{*}(t_{j-1})$, then use $l_{k}=u_{k}$, and determine the total type II error rate. We pick the drift parameter value such that the type II error rate is the desired level, β.

Properties of the beta spending function approach depend on the particular spending function. The O'Brien‐Fleming‐like spending function is

(10) $$\beta_{*}(t)=2\left\{1-\Phi \left(\frac{z_{\beta /2}}{\sqrt{t}}\right)\right\},\;\;0\leq t\leq 1$$

where $z_{\beta /2}$ is the (1−β/2)th percentile of the standard normal distribution function, Φ(·). Function (10) is a very popular choice for alpha spending for efficacy because it spends very little alpha until fairly late in the trial. But the one‐sided alpha level is very small, often 0.025, whereas β is typically between 0.10 and 0.20. For β in this range, the O'Brien‐Fleming‐like beta spending function accelerates earlier. Thus, O'Brien‐Fleming is not nearly as conservative for beta spending as it is for alpha spending.

**FIGURE 4:**
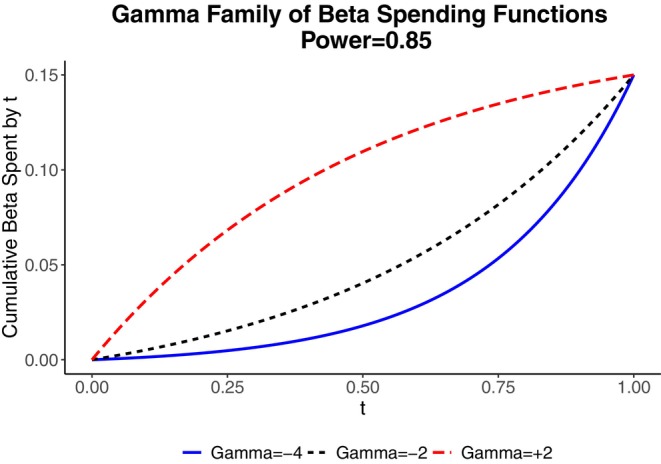
The gamma family of beta spending function with power 0.85 and gamma=−4 (conservative spending), −2 (moderate spending), and +2 (aggressive spending).

There are useful families of beta spending functions indexed by a parameter determining the level of conservatism for early stopping. One popular choice is the Hwang, Shih, and DeCani family (also called the gamma family),

$$\beta_{*}(t)=\beta \left\{\frac{1-\exp(-\gamma t)}{1-\exp(-\gamma )}\right\},\;0\leq t\leq 1$$

where γ can be positive or negative (Figure 4). Small values of γ spend beta conservatively, whereas larger values spend more aggressively. For example, γ=−4 spends beta very gradually. The ACTIV‐1 trial of immune modulators in hospitalized patients with COVID‐19 used the Hwang, Shih, DeCani family with β=0.15 and γ=−2. This spends β more aggressively than the choice γ=−4, but not nearly as aggressively as the choice γ=+2 (Figure 4).

Table 4 shows the lower boundaries for a trial using the Hwang, Shih, DeCani beta spending function with β=0.15 and γ=−4, −2, or +2 for a trial with four equally‐spaced looks. The upper boundary for efficacy is based on the O'Brien‐Fleming‐like spending function with z‐score boundaries 4.3326, 2.9631, 2.3590, and 2.0141 at t=0.25, 0.50, 0.75, and 1. For the conservative choice γ=−4 for beta spending, we can stop for futility at the first analysis only if the z‐score is −1.0607 or less, whereas γ=+2 allows stopping at the first analysis even when results are going in the favorable direction, with a z‐score of 0.2788 All lower boundaries increase over information time, albeit at different rates, and end up at 2.0141 at the end, coinciding with the upper boundary. The price to pay for more aggressive beta spending is a precipitous increase in the drift parameter, resulting in a substantially larger sample size. The conservative choice of γ=−4 requires a drift parameter value of 3.057, only slightly larger than the value 3.00 required for 85% power with no monitoring. In contrast, the aggressive spending choice γ=+2 requires a drift parameter value of 3.535. The squared ratio of drift parameters, ${\left(3.535/3.057\right)}^{2}=1.337$, tells us that the choice γ=+2 requires about a 34% larger sample size than the choice γ=−4.

**TABLE 4:** Lower boundaries, $l_{1},\ldots ,l_{4}$, and drift parameter for the Hwang, Shih, DeCani beta spending function with γ=−4, γ=−2, and +2 for a trial with four equally‐spaced looks and using the O'Brien‐Fleming‐like spending function for efficacy boundaries.

	Number of looks				
	$l_{1}$	$l_{2}$	$l_{3}$	$l_{4}$	Drift
γ=−4	−1.0607	−0.0037	0.9761	2.0141	3.0572
γ=−2	−0.6029	0.3572	1.1927	2.0141	3.1222
γ=+2	0.2788	1.0364	1.5654	2.0141	3.5353

The choice of the beta spending function can be tailored to the particular circumstances. In traditional trials, a relatively conservative value is in order. With a new and dangerous epidemic, the desire to quickly find an effective treatment might lead to a less conservative choice, but opinions vary on the optimal strategy.

We have developed the Beta Spending Function Shiny Web Application to calculate the spend function plots and boundary plots.

### 3.6 Bayesian Futility Methods

We briefly describe two Bayesian approaches to futility analysis. The common thread is that a prior distribution is specified for the true treatment effect, which is then updated to a posterior distribution after seeing interim data. One simple option is to consider stopping for futility if the posterior probability that the true treatment effect exceeds a given cutpoint is less than a specified value. An example of this approach comes from a multiplatform collaboration of three trials in critically ill patients with COVID‐19 randomized to either therapeutic anticoagulation with heparin or pharmacologic thromboprophylaxis according to local usual care [15]. The primary outcome was organ support‐free days up to day 21, where death was assigned a value of −1. A proportional odds model was used, and the Bayesian futility criterion considered stopping if the posterior probability that the odds ratio was less than 1.2 was at least 95%. This threshold was met, and the trial stopped early for futility.

An alternative Bayesian method, first introduced by Spiegelhalter et al. [16], averages conditional power over the posterior distribution of the treatment effect. This is sometimes called *predictive power* [5] and sometimes called *predictive probability of success(PPoS)* [17].

Although Kundu et al. [18] present expressions for PPoS (and other monitoring metrics) in single‐ and two‐arm trials for continuous, binary, and time‐to‐event endpoints, the B‐value formulation provides a single formula that is approximately correct under our Brownian motion framework with a normal prior. Provided that the targeted amount of information remains fixed, we can translate the prior distribution for the treatment effect to a prior distribution for the drift parameter θ of the Brownian motion. If the prior distribution for θ is normal with mean $\theta_{0}$ and variance $\sigma_{0}^{2}$, the PPoS at an interim analysis at information fraction t is

$$PPoS(t)=\Phi \left\{\frac{\left(b-z_{\alpha /2})(1+t\sigma_{0}^{2})+(1-t)(\theta_{0}+b\sigma_{0}^{2}\right)}{\sqrt{\left(1-t)(1+\sigma_{0}^{2})(1+t\sigma_{0}^{2}\right)}}\right\}$$

where b is the interim B‐value (see appendix 3.7.3 of Proschan et al. [5] for a derivation).

Using a prior mean of 0 shrinks the observed treatment effect towards its prior mean. Consequently, if the observed treatment effect is in the wrong direction (i.e., suggests harm), the posterior mean will moderate toward no effect, and PPoS will be higher than conditional power under the observed effect. Similarly, if the observed effect is a strong benefit, PPoS will be lower than the conditional power under the observed effect. Thus, PPoS is a reasonable compromise between computing conditional power under the observed and originally hypothesized effects.

### 3.7 Other Methods

Other futility methods are popular in cancer clinical trials, where it is not uncommon to have a long recruitment period relative to the median survival time. In this case, early termination could prevent a substantial number of people from receiving an ineffective treatment and would save resources. Wieand et al. [19] propose a simple procedure in which a single look occurs when half of the required deaths have occurred; more deaths on the experimental treatment than the control would trigger a serious discussion about stopping for futility. Because there is only a single look, the loss of power is minimal.

Freidlin et al. [20] argue that traditional futility boundaries are too conservative in the middle of a trial and too aggressive toward the end. They suggest starting monitoring for “inefficacy” at the time $t_{0}$ such that results going in the wrong direction (i.e., Z<0 in our parameterization in which positive z‐scores suggest benefit) would imply that a nominal, two‐sided 95% confidence interval for the treatment effect would exclude the effect used to power the trial. They show that

$$t_{0}={\left(\frac{1.96}{z_{\alpha /2}+z_{\beta }}\right)}^{2}$$

where $z_{x}$ denotes the (1−x)th quantile of a standard normal distribution. They consider different strategies:

1. Start monitoring at time $t_{0}$ and stop if $Z(t_{i})<0$ at any interim analysis.
2. Start monitoring at $t=t_{0}$ and stop if $Z(t_{0})<0$. Subsequently, stop at $t_{i}$ if all treatment effects in the nominal 95% confidence interval exclude the hypothesized effect.
3. Similar to 2, but use a sliding scale that makes late stopping more difficult.

Simulations demonstrate only a small loss of power of these procedures and potentially earlier stopping than other procedures. We believe that multiple tools, including those of Wieand et al. [19] and Freidlin et al. [20], can be useful for futility decisions.

## 4 Question 2: Would a Null Result Still Be Meaningful?

When the trial is designed, a power calculation is performed based on assumptions of parameters such as the control event probability for a trial with a binary endpoint, or the variance for a trial with a continuous endpoint. As the trial progresses and monitoring begins, these assumptions can be updated or replaced by estimates from data obtained from the trial itself. Once these estimates are obtained, the original power can be revised (revised power), and if low, it would mean that the trial is unlikely to answer its planned question.

Recall that a low CP (Section 3.1) means that a null result is likely, and a low revised power means that the trial is unlikely to answer the intended question, so low values of both of these quantities signal that not only is it likely that the trial will end in a null result, but that null result will not be meaningful. This is a circumstance in which stopping for futility is indicated. If CP is low, but revised power is high, stopping for futility may not be recommended if the prevailing medical opinion is that the treatment works. If ethical, continuing the trial to overturn that widely held opinion may be helpful.

## 5 Discussion

Futility monitoring should be considered in the design phase of a trial and should consider factors such as phase of the trial, seriousness of the disease, the need to find an effective treatment as quickly as possible, and the effect of futility decisions on the ability to assess secondary endpoints and safety. For example, in an early‐stage trial, a type II error is particularly serious because it could lead to discontinuing further testing of an effective treatment. A multi‐arm trial in a deadly disease like Ebola virus disease might have more aggressive futility guidelines to drop poorly performing arms and continue only promising treatments.

Futility monitoring can stop a doomed trial and allow resources to be reallocated to a more promising agent. Conditional power and revised unconditional power are useful for answering two separate questions: (1) Will the final result be null? and (2) Will a null result be meaningful? If the answers are yes and no, respectively, continuation is pointless. Stochastic curtailment is a guideline such as “consider stopping if conditional power drops below 0.20.” Properties depend on what treatment effect is assumed. The commonly used observed treatment effect is quite variable and can lead to a substantial power loss. The originally hypothesized treatment effect is stable and leads to minimal power loss, but may be unrealistic in light of observed data. Predictive power is a Bayesian alternative that averages conditional power over the posterior distribution of the treatment effect, given the data. Predictive power can be viewed as a compromise between using the originally hypothesized vs. the observed treatment effect.

Reverse conditional power sidesteps the treatment effect issue. Instead of conditioning on the interim result and projecting to the end of the study, we condition on (barely) reaching statistical significance at the end of the trial and ask “What is the probability we would see results at least as unpromising as what we are seeing at the interim analysis?” This probability does not depend on the treatment effect. If such dismal results are extremely unlikely among trials that eventually reach statistical significance, we can be confident that our trial will not be among them.

Predicted interval plots are a useful accompaniment to conditional power that offer an estimation, rather than testing, perspective. We repeatedly simulate and append future data to current results and compute a future confidence interval for the treatment effect. This provides a graphical depiction of conditional power and differentiates between low conditional power caused by very poor interim results or by much higher than expected variability, leading to very wide confidence intervals.

Beta spending functions are analogous to alpha spending functions, but spend type II rather than type I error rates. As with alpha spending, we can spend conservatively or aggressively through different choices of the spending function.

All of the above methods are useful and should be treated as guidelines rather than strict rules. Futility monitoring can slightly decrease the type I error rate because continuation might have led to a statistically significant benefit. Some people have advocated recovering the lost type I error rate by modifying efficacy boundaries to account for futility monitoring. This practice should be avoided because the type I error rate would be controlled only if the futility guideline is strictly followed. Even efficacy boundaries are guidelines, rather than rules, in the sense that other factors almost invariably come into play when making stopping decisions. Futility guidelines are usually considered even less binding. The final decision almost always depends on numerous other factors, such as whether (1) the trial will still provide useful information about safety or secondary endpoints, (2) enrollment has been completed and the cost of continuation is low, (3) the disease is mild and the intervention is in widespread use, so continuation might be warranted to demonstrate that it does not work. Abandoning a disappointing trial has consequences. Weigh the results of the above statistical tools with extra‐statistical considerations before making the final decision.

## 6 Disclosure

The authors have nothing to report.

## Conflicts of Interest

The authors declare no conflicts of interest.

## Figures and Tables

**FIGURE B1:**
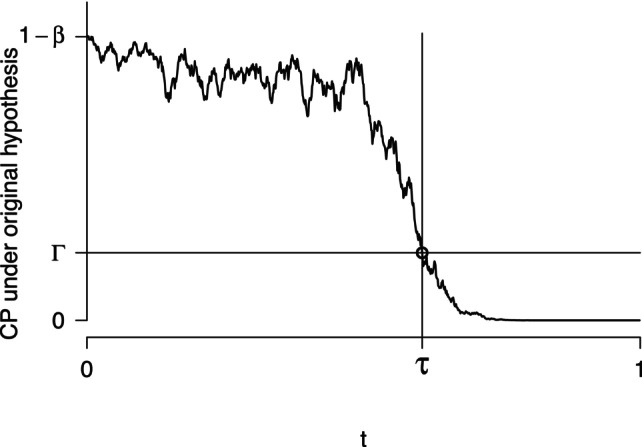
Continuous monitoring under the original hypothesis using a CP threshold of Γ (horizontal line). CP(t) is continuous and τ is the time of first reaching CP level Γ, with τ=2 if CP level Γ is never reached.

## Data Availability

The authors have nothing to report.

## Appendix A Formulas for B‐Values and Z‐Scores

This appendix heuristically derives formulas for B‐values and Z‐scores. Recall that the B‐value at information fraction t behaves like $S_{I}/\sqrt{I_{\text{end}}}$, where I and $I_{\text{end}}$ are the interim and final information, $t=I/I_{\text{end}}$ is the information fraction, and $S_{I}=I\hat{\delta }$ behaves like a sum of I iid. N(δ,1) observations. Accordingly,

(A1)
$$\begin{array}{l} E\{B(t)\}=\frac{E(S_{I})}{\sqrt{I_{\text{end}}}}=\frac{I\delta }{\sqrt{I_{\text{end}}}}=\left(\sqrt{I_{\text{end}}}\;\delta \right)\left(\frac{I}{I_{\text{end}}}\right)=\theta t \end{array}$$

where θ=E{Z(1)} is the expected z‐score at the end of the trial. Also, E{B(1)−B(t)}=θ·1−θ·t=θ(1−t).

The variance of B(t) is

(A2)
$$\begin{array}{l} \mathrm{var}\{B(t)\}=\frac{\mathrm{var}(S_{I})}{I_{\text{end}}}=\frac{I}{I_{\text{end}}}=t \end{array}$$

Also, B‐values inherit the independent increments property from the corresponding sums, so $B(t_{2})-B(t_{1})$ is independent of $B(t_{1})$ for $t_{1}\leq t_{2}$. That provides an easy way to deduce the variance of $B(t_{2})-B(t_{1})$:

(A3)
$$\begin{array}{ll} t_{2} & =\mathrm{var}\{B(t_{2})\}=\mathrm{var}\{B(t_{1})+B(t_{2})-B(t_{1})\}\\ & =\mathrm{var}\{B(t_{1})\}+\mathrm{var}\{B(t_{2})-B(t_{1})\}\;\text{(independent increments)}\\ & =t_{1}+\mathrm{var}\{B(t_{2})-B(t_{1})\} \end{array}$$

from which $\mathrm{var}\{B(t_{2})-B(t_{1})\}=t_{2}-t_{1}$. Alternatively, we can compute $\mathrm{var}\{B(t_{2})-B(t_{1})\}$ as $\mathrm{var}\{B(t_{2})\}+\mathrm{var}\{B(t_{1})\}-2\;\text{cov}\{B(t_{1}),B(t_{2})\}$ and use the fact that

$$\text{cov}\{B(t_{1}),B(t_{2})\}=t_{1}\;\text{for}\;t_{1}\leq t_{2}$$

Properties of z‐scores follow from those of B‐values and the relationship $Z(t)=B(t)/\sqrt{t}$. For example, for $t_{1}\leq t_{2}$,

(A4)
$$\begin{array}{ll} \text{cov}\{Z(t_{1}),Z(t_{2})\} & =\text{cov}\left\{\frac{B(t_{1})}{\sqrt{t_{1}}},\frac{B(t_{2})}{\sqrt{t_{2}}}\right\}\\ & =\left(\frac{1}{\sqrt{t_{1}t_{2}}}\right)\text{cov}\{B(t_{1}),B(t_{2})\}=\frac{t_{1}}{\sqrt{t_{1}t_{2}}}\\ & =\sqrt{t_{1}/t_{2}} \end{array}$$

## Appendix B Proof of CP Bound on Power Loss

Let CP(t) denote conditional power under the original hypothesis at information fraction t. At t=0, there is no data, and CP(0) is just the unconditional power of the trial with no monitoring, namely 1−β. The stochastic curtailment rule stops for futility at time t if CP(t)≤Γ. If we monitor continuously, CP(t) traces out a random, continuous curve (Figure B1). A key fact is that for Z(1)<1.96, the CP threshold Γ is reached for some t<1. This follows from 3 facts: (1) CP(t) is continuous, (2) CP(0)=1−β, which exceeds Γ, and (3) CP(t) approaches 0 as t→1 if Z(1)<1.96. Consequently, {Z(1)<1.96} is equivalent to {Z(1)<1.96}∩{τ<1}, where τ is defined as the first time CP(t)≤Γ, or τ=2 if CP(t)>Γ for all t<1. Another key fact is that, by definition, if τ=t<1, then CP(t)=Γ. Let F(t) denote the distribution function of τ. Then

(B1)
$$\begin{array}{ll} \beta & =P_{\theta }\{Z(1)<1.96\}=\int_{0}^{1}P_{\theta }\{Z(1)<1.96\;|\;\tau =t\}dF(t)dt\\ & =\int_{0}^{1}(1-\Gamma )dF(t)=(1-\Gamma )P(\tau <1),\;\text{so}\\ P(\tau <1) & =\frac{\beta }{1-\Gamma } \end{array}$$

This shows that for continuous monitoring, the type II error rate, P(τ<1), is exactly β/(1−Γ).
